# Real-Time Spatiotemporal Seismic Fragility Assessment of Structures Based on Site-Specific Seismic Response and Sensor-Integrated Modeling †

**DOI:** 10.3390/s25165171

**Published:** 2025-08-20

**Authors:** Han-Saem Kim, Taek-Kyu Chung, Mingi Kim

**Affiliations:** 1 Department of Civil and Environmental Engineering, Dongguk University, Seoul 04620, Republic of Korea; hansaem@dgu.ac.kr; 2 Department of Civil and Environmental Engineering, Seoul National University, Seoul 08826, Republic of Korea; 3 Disaster Management Research Center, Seoul Institute, Seoul 06756, Republic of Korea

**Keywords:** real-time assessment, structure seismic fragility, site-specific seismic response, GIS, sensor-informed triggering protocol

## Abstract

Earthquake hazards, such as strong ground motion, liquefaction, and landslides, pose significant threats to structures built on seismically vulnerable, loose, and saturated sandy soils. Therefore, a structural failure evaluation method that accounts for site-specific seismic responses is essential for developing effective and appropriate earthquake hazard mitigation strategies. In this study, a real-time assessment framework for structural seismic susceptibility is developed. To evaluate structural susceptibility to earthquakes, seismic fragility functions are employed as thresholds for structural failure and are linked to a geotechnical spatial grid that incorporates correlation equations for seismic load determination. The real-time assessment consists of the following procedures. First, the geotechnical spatial grid is constructed based on the geostatistical method to estimate the site-specific site response to be correlated with the earthquake hazard potential. Second, the peak ground accelerations are determined from seismic load correlation and assigned to the geotechnical spatial grid. Third, the damage grade of structure is determined by calculating the failure probabilities of defined damage levels and integrating the geotechnical spatial grids for the target structure in real time. The proposed assessment was simulated at Incheon Port, South Korea, using both an actual earthquake event (the 2017 Pohang Earthquake) and a hypothetical earthquake scenario.

## 1. Introduction

Earthquakes are among the most devastating natural disasters, causing significant loss of life, infrastructure damage, and economic disruption globally. The frequency and magnitude of seismic events vary considerably by region, with certain areas experiencing a higher concentration of high-intensity earthquakes due to tectonic activity. Recent studies have emphasized the critical role of local soil characteristics in influencing earthquake-induced damage. For example, near-fault ground motions exhibit different responses depending on the underlying soil type, which can significantly affect the seismic behavior of buildings and infrastructure [1,2]. In particular, soft soils have been shown to amplify seismic waves, leading to greater structural damage compared with more stable rocky foundations [2,3]. Furthermore, variations in soil suction and overburden thickness directly influence ground motion characteristics, making it essential to account for these factors in seismic hazard assessments [4]. Understanding the correlation between soil properties and seismic response is crucial for improving building codes and mitigating earthquake risks, particularly in urban areas with complex geological conditions [5].

Recently, the increasing frequency of earthquake events has highlighted the growing need for seismic studies in South Korea, where geotechnical hazards pose significant threats to structures in ports and urban areas built on loose, saturated sandy soils. Consequently, the evaluation of geotechnical earthquake hazards has gained increasing emphasis in South Korea. Seismic disaster management and mitigation for building structures are required to establish effective and appropriate strategies to reduce earthquake hazards. However, these tasks are complex and require substantial resources and detailed analysis [6,7,8,9]. Their complexity requires the use of a systematic methodology based on a computer-aided system, such as the geographic information system (GIS) tool [4]. In addition, establishing a database and estimating the spatial extent of seismic vulnerability necessitate the use of a wireless network system (WNS) connected to seismometers.

The fragility of a structure or component is defined as the conditional frequency of failure given a specific value of the response parameter. The response parameter x can represent forces acting on a component or structure due to wind, earthquake-induced stress, or other external forces. The conditional frequency of failure $F_{0}$ given $x_{0}$ is the fragility of the component at that level. In other words, the fragility curve is the cumulative distribution function of the component’s capacity to withstand the imposed stress x. In many seismic risk studies, the response parameter x is the peak horizontal ground acceleration measured in units of g, the gravitational acceleration. Furthermore, the fragility curve usually has a lognormal distribution (other distributions have also been considered, like the truncated lognormal distribution and the normal distribution).

The time-variant reliability problem in broad terms is the problem of computing the probability that a nonlinear system with random properties exposed to random, time-varying actions ceases to satisfy a number of requirements, whose definition is also subject to uncertainty. In seismic reliability analysis, this probability is typically conditioned on an intensity measure of the input ground motion, referred to as the fragility function. To calculate the unconditional probability that a structure will fail at a given site and time, seismic intensity uncertainty is incorporated using the hazard function (the complementary cumulative distribution function (CDF) of intensity). By convolving the hazard function’s derivative with the fragility function, seismic risk is determined.

A large number of methods have been proposed to compute fragility functions in the last 20 years, ranging from expert judgment [10] to data analysis on observed damages [11,12] and fully analytical approaches, such as, for example, in [13,14,15,16,17]. General reviews can be found, for example, in [18,19]. A feature common to most of the analytical approaches is the use of a reduced number of simulations to compare probabilistically the maximum structural responses with the corresponding capacities. The primary differences lie in how they balance cost and accuracy, particularly in their ability to economically account for all aspects of the reliability problem. These latter include the following:

1. The possibility of the structure collapsing in more than one failure mode (the system reliability problem), as is common for civil engineering structures such as multistory buildings and bridges;
2. The dependence among the possible failure modes;
3. The uncertainty in the capacity of the structure due to the approximate nature of the models and the variability in the system parameters;
4. The influence on the dynamic response of the variability in the system parameters.

In this study, we developed a systematic procedure for real-time structure seismic fragility assessment that consists of three functional modules within a database (geotechnical spatial grid construction, real-time seismic load determination, and structure fragility evaluation). The datasets for real-time earthquake hazard assessment consist of geographic, geotechnical, structural, and seismic monitoring data for the target site.

First, the geotechnical spatial grid is constructed based on the geostatistical method to estimate the site-specific site response to be correlated with the earthquake hazard potential. Second, the peak ground accelerations are determined from seismic load correlation and assigned to the geotechnical spatial grid. Third, the damage grade of the structure is determined by calculating the failure probabilities of defined damage levels and integrating the geotechnical spatial grids for the target structure in real time. A simulation of the proposed assessment was specifically conducted at Incheon Port, South Korea, using an actual earthquake event (2017 Pohang Earthquake) and a virtual earthquake scenario.

## 2. Methods

### 2.1. Framework Architecture

**Figure 1:**
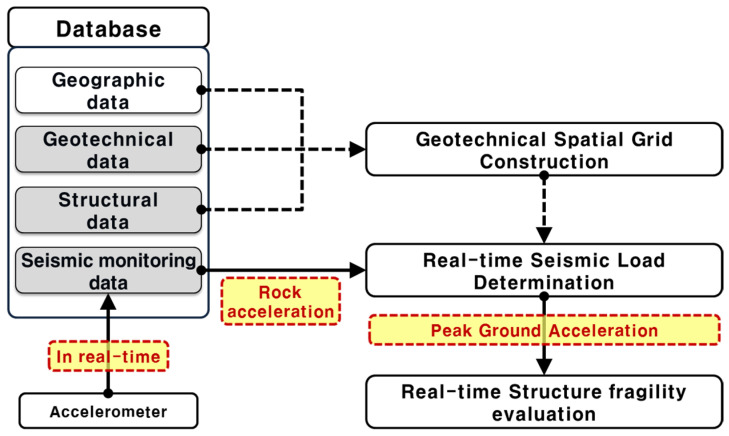
Computer-based framework architecture for the real-time assessment of structural seismic fragility (modified from Kim et al. [4]).

The proposed real-time spatiotemporal assessment framework for structural seismic fragility comprises three interdependent modules (geotechnical spatial grid construction, real-time seismic load determination, and structural fragility evaluation) integrated within a geospatial database environment. These modules are structured to operate in both pre-event preparation and post-event real-time assessment modes (Figure 1) [4].

In the first module, a three-dimensional (3D) geotechnical spatial grid is constructed based on geostatistical interpolation techniques. Ordinary kriging is employed using a spherical variogram model to interpolate borehole-derived soil classification and dynamic properties. This module incorporates geographic coordinates, geotechnical profiles (classified using the Unified Soil Classification System (USCS)), dynamic soil properties (shear wave velocity, shear modulus, and damping ratio), and structural attributes of the target infrastructure.

In the second module, seismic load correlations are developed by performing 1D equivalent-linear site response analyses using ProShake, employing recorded and synthetic ground motions across multiple rock acceleration levels (0.04 g to 0.50 g). For each geotechnical grid cell, peak ground acceleration (PGA) values are estimated from bedrock acceleration using a nonlinear regression model. The Box–Lucas exponential model was selected due to its capacity to capture soil nonlinearity. Correlation coefficients (a, b) for each soil layer were calibrated by minimizing the root mean square error (RMSE) between simulated and observed surface PGA values, with R^2^ values exceeding 0.85 in most cases.

The third module applies seismic fragility curves to evaluate the structural failure probability. PGA values derived in real time are used as input intensity measures. Structures above soil layers utilize site-specific PGAs, while structures founded on bedrock use direct rock outcrop acceleration. Fragility curves are modeled as cumulative lognormal distributions, with median and dispersion parameters derived from empirical studies. Damage states are categorized as Slight, Moderate, Extensive, or Complete, with structural failure defined as exceeding a 50% probability of a damage state.

### 2.2. Event Triggering and Real-Time Data Exchange Between Seismic Monitoring and Fragility Assessment Systems

**Figure 2:**
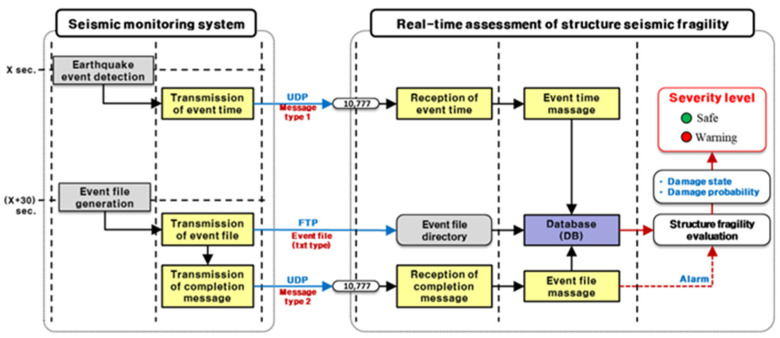
Schematic flow of the real-time transmission and trigger protocol of the earthquake event files from the integrated port earthquake alert system [2].

Upon seismic event detection, the monitoring system immediately generates a trigger message containing the event ID, timestamp, and acceleration time history. This message is transmitted via a UDP-based Type I protocol to the real-time structural fragility assessment platform. Concurrently, the corresponding acceleration file is created and transmitted through an FTP-based protocol to the designated event file directory on the assessment server. Following successful data delivery, a completion message is sent using a UDP-based Type II protocol, enabling synchronization across all components (Figure 2).

The real-time assessment server—equipped with an Oracle-based spatially linked relational database—automatically ingests both the event metadata and raw acceleration files. These inputs initiate a sequence of automated processes including event parsing, seismic load estimation, and structural fragility evaluation. All intermediate steps, such as event time messaging and file registration, are timestamped and verified to ensure data integrity and synchronization.

This tightly integrated system architecture enables the ingestion, processing, and visualization of seismic hazard information to be completed within 50 s of the earthquake’s occurrence. The modular framework consists of sensor instrumentation (triaxial strong-motion accelerometers at the ground surface and structure base levels), digital data acquisition systems, automatic triggering modules, redundant data communication channels, and an uninterrupted power supply (UPS) for resilience during major events. All sensing units are GPS-synchronized to maintain sub-second accuracy in event time tagging, which is critical for correlating bedrock motion with the structural response. Its ability to deliver high-fidelity seismic hazard and structural fragility information within 50 s of an event’s occurrence enables informed and timely decision-making, especially in critical coastal infrastructure systems.

Seismic data are visualized through a GIS-based interface—currently implemented at key Korean ports such as Busan—enabling real-time system status monitoring and damage visualization. The generated fragility assessments are site-specific and use pre-stored geotechnical spatial grids and regression-based PGA estimates to produce hazard maps categorized by severity level (e.g., Safe, Warning).

Unlike general-purpose seismic networks, this system is explicitly designed to support spatiotemporally resolved evaluations of structural integrity by directly linking bedrock acceleration monitoring with surface motion prediction and fragility curve modeling. Its scalable and modular design supports rapid deployment and customization across a wide range of coastal infrastructure systems, offering a robust and resilient backbone for earthquake disaster management.

In summary, the described architecture establishes a reliable and efficient end-to-end workflow from seismic monitoring to real-time structural fragility assessment. Its ability to deliver high-fidelity hazard information within seconds enhances the decision-making capacity for emergency responses and post-earthquake structural verification in high-risk regions.

### 2.3. Geotechnical Spatial Grid Construction

**Figure 3:**
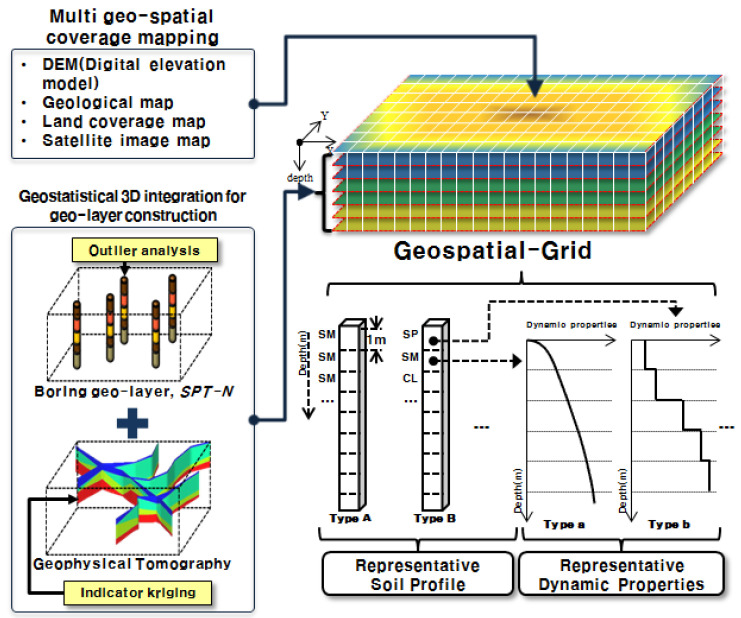
Procedure for geotechnical grid construction integrating multi-layer surface maps, borehole/SRT data, kriging-based modeling, and dynamic property assignment [2,4].

To enable site-specific assessment of seismic fragility, a geotechnical spatial grid is constructed using a geostatistical modeling framework that integrates surface coverage data, borehole logs, and geophysical imaging (Figure 3). This spatial database stores stratified soil layers and the corresponding dynamic properties in a 3D structure, providing foundational input for ground response and real-time hazard evaluation modules.

Spatial grid construction begins with the incorporation of multiple surface layers, including digital elevation model (DEM), geological map, land cover classification, and satellite imagery layers. These datasets are spatially aligned using a unified coordinate system and digitized to form the upper boundary of the grid. The target area is discretized into uniform volumetric cells measuring 5 m × 5 m × 1 m to ensure a resolution suitable for structural-scale seismic modeling.

Subsurface information is obtained from borehole data and standard penetration test (SPT-*N*) results collected across two designated zones. Since these data provide only one-dimensional profiles with limited spatial coverage, three-dimensional kriging interpolation is employed to estimate the lateral and vertical distribution of soil types. Prior to interpolation, outlier analysis is conducted to exclude anomalous data and enhance the statistical robustness of the model [16]. All soil types are classified based on the USCS, and primary stratigraphic units—such as fill, sandy, clayey, and weathered soils—are delineated accordingly [20,21,22,23].

In areas with sparse borehole coverage or topographic complexity, such as boundary zones or hill slopes, additional geophysical tomography data are incorporated using indicator kriging to supplement the interpolated results [3,14,15]. This hybrid approach reduces the potential for misclassification, such as predicting rock as soil, and improves the reliability of stratigraphic predictions at the domain edges.

For each classified soil unit in the grid, dynamic geotechnical properties are assigned, including shear wave velocity (*V_S_*), normalized shear modulus, and material damping ratio. These values are obtained from in situ testing data and literature-based correlations. In particular, *V_S_* is estimated by interpolating SPT-*N* values through ordinary kriging and applying the empirical correlation proposed by Sun et al. [17], thereby linking field measurements to seismic response properties.

The final geotechnical spatial grid also includes a surface coverage layer derived from the DEM and thematic maps. These topographic and thematic datasets, such as geological and land use maps, are digitized and aligned with the geotechnical layers to allow for comprehensive spatial querying. All information is stored in a GIS-compatible format, enabling the visualization and retrieval of site-specific data under real-time processing conditions.

Once the geospatial grid is constructed, it is integrated into the central hazard analysis system. During an earthquake, recorded acceleration signals are transmitted to the system, and site-specific ground conditions—stored in the geotechnical spatial grid—are retrieved in real time to compute the site response and structural fragility. This spatial modeling approach significantly improves the resolution and reliability of seismic hazard predictions, especially in data-scarce regions, and establishes a robust basis for adaptive and scalable disaster risk assessment.

### 2.4. Real-Time Seismic Load Determination

To support immediate estimation of seismic demand following an earthquake, the proposed framework incorporates a real-time seismic load determination module that is directly triggered by seismic event detection through the sensor-based monitoring network described in Section 2.2. Upon receipt of real-time ground motion data—specifically rock outcrop acceleration values transmitted via the sensor network—the module utilizes pre-established nonlinear regression models to estimate corresponding peak ground accelerations (PGAs) at the surface across the geotechnical spatial grid.

These regression models were calibrated in advance through one-dimensional site response analyses and are stored within the database-linked spatial grid. By referencing these empirical relationships, the system eliminates the need for computationally intensive site response analysis during the event, which is generally infeasible under strict time constraints. As a result, site-specific seismic demand can be rapidly computed and supplied to the structural fragility evaluation module within seconds of event occurrence, thereby enabling spatiotemporally synchronized hazard assessment across the monitoring and response systems [24].

The correlation models were established through a series of site response analyses performed in advance using the equivalent-linear method implemented in ProShake [25], a widely accepted 1D site response program. Input ground conditions for the analyses were defined based on the geotechnical spatial grid, including soil classification and dynamic soil properties such as shear wave velocity, shear modulus, and damping ratio.

**Figure 4:**
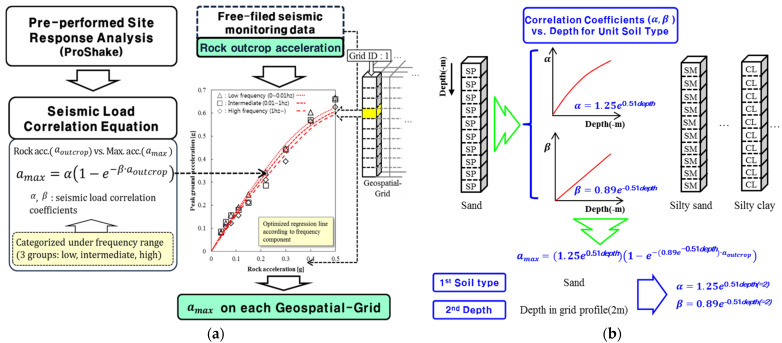
(**a**) Procedure for real-time seismic load determination using pre-performed site response analysis and nonlinear regression modeling to correlate bedrock acceleration with surface ground motion [24,26]. (**b**) Normalization of seismic load correlation coefficients based on soil classification and depth using exponential fitting.

To represent a wide range of seismic shaking intensities, nine discrete levels of input rock outcrop acceleration were considered, ranging from 0.04 g to 0.50 g. The ground motion inputs included three representative acceleration time histories: Hachinohe (short-period dominant), Ofunato (long-period dominant), and an artificial wave with broadband characteristics [24,26]. For each geospatial grid cell, 27 site response simulations were conducted (9 acceleration levels × 3 input motions), and corresponding surface PGA values were calculated. These data were then used to develop nonlinear regression models describing the correlation between the input rock acceleration $a_{rock}$ and the resulting layer peak acceleration $a_{max}$ (Figure 4).

The regression model adopts the Box–Lucas exponential function [27]:

(1) $$a_{max}=\alpha \left(1-e^{-\beta \cdot a_{rock}}\right)$$

where $a_{max}$ denotes the peak ground acceleration of each layer, $a_{rock}$ represents the measured rock acceleration, and α and β are correlation coefficients, respectively. The correlation equations are incorporated into the geotechnical spatial grid, enabling real-time determination of PGA from the measured rock acceleration [3].

To streamline this process for broader-scale applications, and to reduce the computational demand for each individual cell, the regression models were further normalized by soil classification and depth using generalized correlation coefficient functions. Specifically, the variation in α and β with depth was statistically modeled for each USCS soil type using exponential regression (Figure 4) [4,28]:

(2) $$\alpha =1.25\cdot e^{0.51\cdot depth},\;\beta =0.89\cdot e^{-0.51\cdot depth}$$

As a result, once the soil type and depth are identified from the geotechnical grid, the corresponding coefficients α and β can be immediately computed and applied. This two-step approach—soil-type first, depth second—ensures rapid retrieval of correlation parameters and facilitates scalable real-time seismic load estimation across complex infrastructure networks. This formulation enables rapid, automated calculation of the PGA at each grid cell using only the measured rock outcrop acceleration and local soil profile. The derived correlation equations are stored in the geotechnical spatial grid database and are invoked automatically during real-time operation. When a seismic event occurs, free-field accelerometers measure the rock outcrop acceleration in real time. This input is passed into the grid-based correlation model, returning site-specific PGA values for each geospatial cell, which are then used as the seismic demand in fragility assessment.

### 2.5. Real-Time Structure Fragility Evaluation

**Figure 5:**
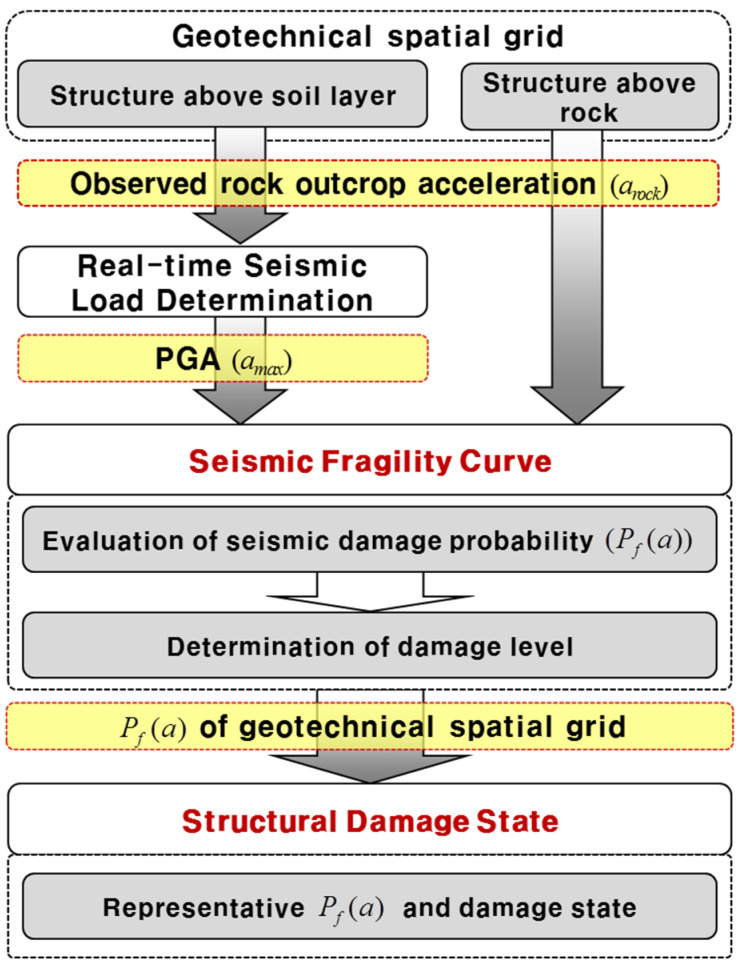
Procedure for real-time assessment of structural fragility assigned by the geotechnical spatial grid and its site-specific characterization of the seismic response (modified from Kim et al. [4]).

To evaluate structural vulnerability in real time during seismic events, a fragility-based assessment framework was developed that directly links observed or derived ground motion parameters to a structural damage probability. This approach leverages the geotechnical spatial grid system to assign a site-specific seismic loading, which is then propagated through predefined fragility functions to estimate the structural response (Figure 5).

Structures are broadly classified into two types based on subsurface conditions: those founded on soil layers and those directly on rock. For soil-founded structures, the fragility assessment is based on the site-amplified peak ground acceleration (PGA) derived from the seismic load correlation model. For rock-founded structures, the input motion is the observed rock outcrop acceleration directly transmitted from free-field accelerometers.

The seismic fragility function expresses the conditional probability of structural damage exceeding a certain level, given a specified seismic intensity measure. While several indices, such as spectral acceleration (Sa) and spectral displacement (Sd), are common, PGA was adopted in this study for its simplicity and compatibility with the spatial correlation model. The general form of the fragility function is:

(3) $${PF}_{ij}=PROB\left[D\geq C_{i}|{EQ}_{j}\right]$$

where ${PF}_{ij}$ is the probability of exceeding damage level i for an earthquake with an intensity of j, D denotes the effect of the load by the earthquake, and $C_{i}$ is the strength of the structure for damage level i.

Appropriate indices representing structural damage should be selected. The fragility function follows a cumulative lognormal distribution and is typically expressed as:

(4) $$P_{f}(s)=\emptyset \left[\frac{\ln s-\ln\bar{s}}{\beta }\right]$$

where $P_{f}(s)$ is the damage probability of the structure at s, ∅[] denotes a Gaussian cumulative lognormal distribution function, $\bar{s}$ is the median of the PGA at the ground surface, s is the PGA of the earthquake, and β is the standard deviation of the log value of the PGA at the ground surface [4].

**Figure 6:**
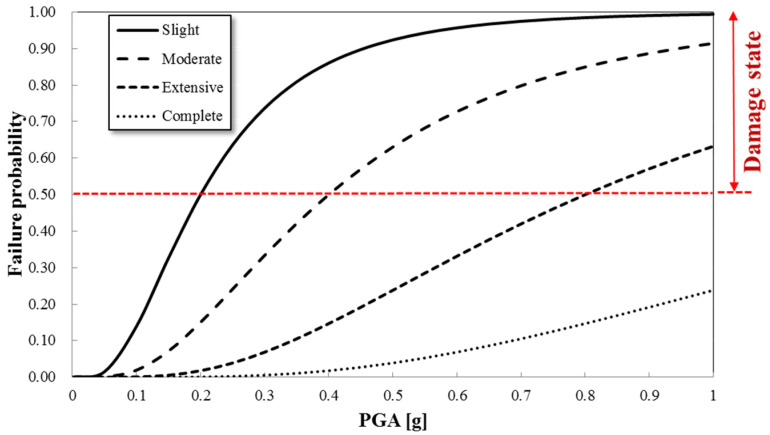
An example of a fragility curve and damage state for an unreinforced concrete structure modified from [29].

Using the real-time seismic load (either the $a_{rock}$ or grid-based $a_{max}$), the corresponding damage probability $P_{f}(s)$ is calculated per geospatial grid cell. The damage grade is then classified based on whether the exceedance probability at each damage level surpasses a predefined threshold (typically 50%). If multiple damage states exceed this threshold, the highest severity state is assigned as the structural condition. For example, if the probabilities for ‘Slight’ and ‘Moderate’ damage are both above 50%, the structure is assigned a ‘Moderate’ grade [4,24]. Figure 6 illustrates a representative fragility curve for unreinforced concrete structures, where the relationship between PGA and various damage states (Slight, Moderate, Extensive, and Complete) is plotted. The red dashed line indicates a real-time evaluation of the PGA, which intersects the fragility curves to identify corresponding damage probabilities.

The final structural damage classification for a facility is determined by the areal dominance of critical damage grades across the geotechnical spatial grid. That is, if more than 50% of the total structural footprint is occupied by cells with a damage state exceeding ‘Moderate’, the overall structural state is labeled a ‘Failure’. This spatial aggregation approach allows for a coherent representation of distributed damage and supports decision-making for emergency responses and retrofitting prioritization.

Unlike traditional post-event assessments, this framework enables automated, near-instantaneous estimation of seismic damage by fusing real-time sensor input with pre-parameterized fragility curves and spatial ground condition databases. The methodology is scalable to urban and port infrastructure, and its rule-based interpretation logic is compatible with national seismic monitoring systems.

## 3. Results

### 3.1. Simulation Conditions

**Figure 7:**
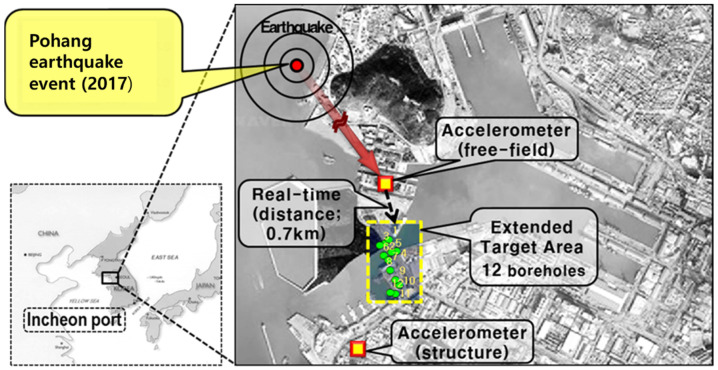
Simulation test conditions for earthquake scenarios for the Incheon Port in South Korea (modified from Kim et al. [4]).

The real-time assessment framework for seismic fragility evaluation was applied to the Incheon Port in South Korea, using actual and hypothetical earthquake events to verify the proposed framework’s applicability. Due to the rapid growth of international trade and travel, seaports and harbors in coastal areas have become critical for the sustainability of local and regional industries and economies. Figure 7 illustrates the target area, which includes the real-time transmission of seismic monitoring data during earthquake events. Two accelerometers were installed at the test site to monitor the free-field ground motion (at the lock) and structural motion (at the passenger terminal) [4,24].

**Figure 8:**
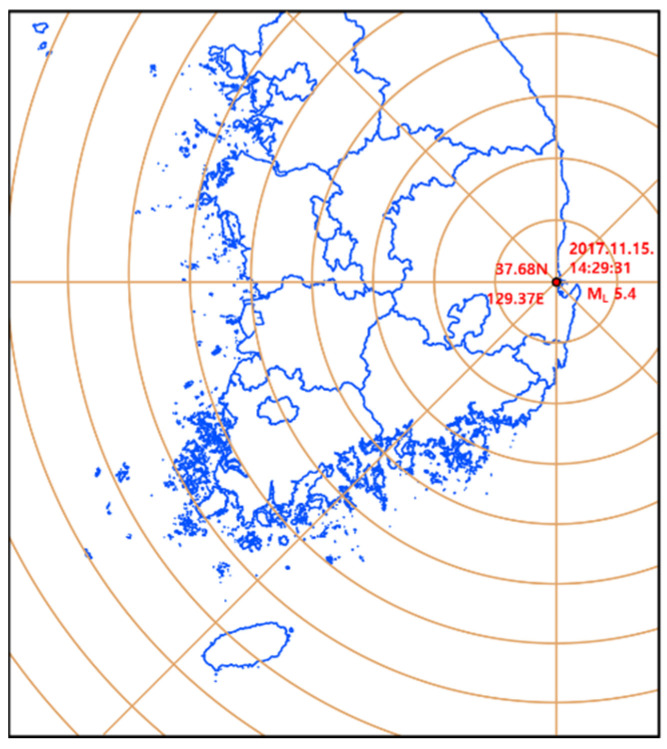
Pohang earthquake event map [30].

In 2017, a significant earthquake occurred in Pohang, South Korea, and seismic monitoring data were collected at permanent and temporary stations. The Pohang earthquake, with a magnitude of 5.4, was the second-largest earthquake recorded since seismic monitoring began in South Korea in 1978. The simulation conditions for the Pohang earthquake were obtained from [30] (Figure 8 and Table 1). However, since no major earthquake events have been recorded in the Incheon Port region, a hypothetical earthquake scenario was also simulated using monitored records from the Pohang seismic monitoring station (PHA2, administrated by the Korean Meteorological Administration), which is the station closest to the Pohang earthquake’s epicenter [30]. For this hypothetical event, input rock outcrop accelerations were determined using a ground motion attenuation relationship [31], which reflects ground shaking trends in South Korea. The attenuation equation is expressed as:

(5) $$\ln a=c_{0}+c_{1}M+c_{2}\ln R+c_{3}R$$

where a denotes the rock outcrop acceleration, M (=5.4) represents the magnitude of the Pohang earthquake, R (=50 km) is the epicentral distance, and $c_{1}\;(=0.4854)$, $c_{1}\;(=1.2)$, $c_{2}$ (=−0.8416), and $c_{3}\;(=-0.61)$ are attenuation coefficients, respectively [31].

Thus, a was defined as the rock outcrop acceleration for the recorded earthquake event and hypothetical earthquake scenario. The a recorded at Incheon Port station (located approximately 0.7 km from the target site (Figure 7) and over 400 km from the epicenter) during the Pohang earthquake event was 0.032 g, whereas the PGA estimated using the ground motion attenuation equation for the hypothetical scenario was 0.138 g (Table 1). These respective values were incorporated into the event-triggering mechanism and the real-time data exchange framework established between the seismic monitoring system and the seismic fragility assessment model.

The target site is part of the Incheon Port passenger terminal, and 12 borehole datasets were stored in the database. Based on the design report and satellite imagery, the extended target area measured 73,600 m^2^ (160 m from west to east and 460 m from north to south). Ground coverage at the port has evolved over the past 40 years due to dredging and soil reclamation efforts [28]. Consequently, the soil conditions have changed over time, with reclaimed areas previously covered by water.

**Figure 9:**
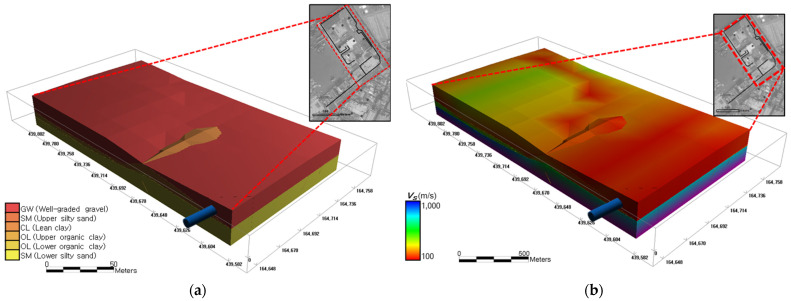
Three-dimensional spatial distribution of (**a**) the depth to bedrock and (**b**) *V_S_* based on the geotechnical spatial grid for the study area of Incheon Port.

The passenger terminal (marked with a dotted black line in Figure 9) was constructed using unreinforced concrete. Fragility functions for the unreinforced concrete frame were determined based on the real-time seismic fragility evaluation method. The fragility functions used were derived from unreinforced masonry infill walls [29] (dotted red lines in Figure 4). The damage levels were categorized as ‘Slight’, ‘Moderate’, ‘Extensive’, and ‘Complete’. Table 2 provides the median and standard deviation of these fragility functions. Structural failure was assumed when the probability of exceeding a damage level reached 50% or higher.

### 3.2. Geotechnical Characteristics of the Seismic Response

To support the real-time seismic fragility assessment framework, a high-resolution three-dimensional geotechnical spatial grid was constructed for the target port facilities using GIS-based spatial interpolation techniques. The spatial grid modeling was based exclusively on borehole data collected from two designated facility zones within the port. The observation sites were geo-referenced using GPS coordinates [2,32], ensuring spatial consistency across all measured geotechnical parameters.

For each zone, an experimental variogram was modeled using an exponential function, followed by the application of ordinary kriging to interpolate the subsurface geotechnical layers. The resulting three-dimensional spatial grid consisted of cells measuring 5 m × 5 m × 1 m, enabling detailed representation of the subsurface variability. A total of 68 grid cells, each with 20 m surface spacing, were selected to cover the primary study area (19,200 m^2^), while the surrounding buffer zone (totaling 73,600 m^2^) provided contextual interpolation support to minimize edge effects [4,24].

To visualize the multi-scale spatial distribution of subsurface strata with respect to both lateral extent and vertical depth, a custom 3D subsurface rendering program was developed using OpenGL functions. This visualization approach accounted for the non-uniform scaling conditions between horizontal and vertical axes, enabling intuitive and accurate rendering of subsurface features (Figure 9).

Additionally, spatial and geometrical information of the sewer pipelines located beneath the target facility zones was superimposed on the geotechnical spatial grid. Through this integration, local geotechnical zoning adjacent to pipelines was extracted and analyzed. As illustrated in Figure 9a, approximately 65% of the sewer alignment within Zone 1 is embedded in sandy soil layers, while the remaining 35% intersects clayey deposits. In contrast, for Zone 2, the sewer alignment spans sandy soil (42%), clayey soil (25%), and gravel (33%) layers. These spatially resolved lithological profiles, extracted in three dimensions relative to ground surface elevation, provided foundational input for evaluating localized seismic response characteristics and potential ground failure scenarios.

To facilitate site response analysis, *V_S_* were estimated for each grid cell using the empirical correlation proposed by Sun et al. [17], where *V_S_* = 65.64 × *N*^0.407^. This correlation was applied to the spatially distributed standard penetration test (SPT) *N*-values stored within the grid, producing a three-dimensional distribution of *V_S_* values across the study area, as shown in Figure 9b. These *V_S_* profiles were used as input parameters for the dynamic site response analysis, ensuring spatial coherence between measured geotechnical properties and the seismic hazard modeling.

In summary, the developed 3D geotechnical grid and associated subsurface visualization framework not only provided a detailed understanding of site-specific subsurface conditions but also enabled their direct integration into the real-time seismic fragility assessment pipeline. The spatial resolution and geological fidelity of this model support accurate, site-adaptive earthquake impact assessments for critical port infrastructure.

### 3.3. Seismic Fragility of Port Structures 

**Figure 10:**
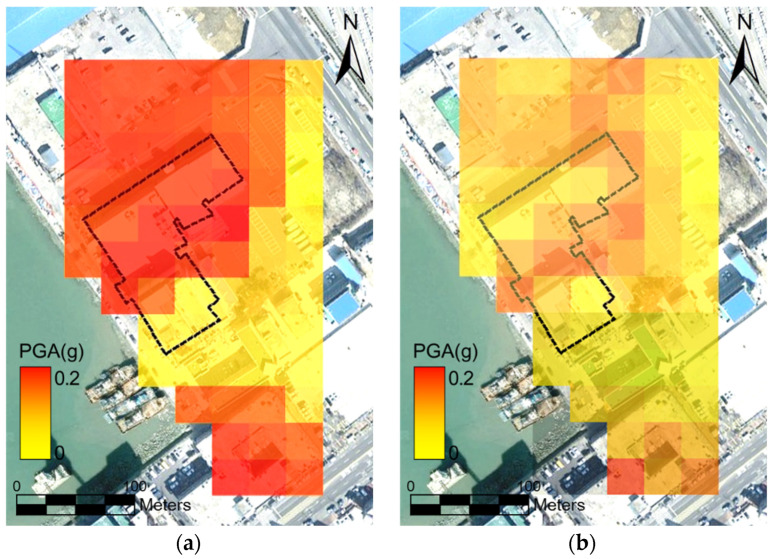
Spatial distribution of the PGA for the Incheon Port study area. (**a**) Hypothetical Pohang earthquake scenario and (**b**) Pohang earthquake event.

The proposed framework was applied to two earthquake scenarios in the study area using geotechnical spatial grids representing the soil profile. Seismic load correlation equations assigned to the geotechnical spatial grid were used to determine the peak ground acceleration (PGA) in real time [4,24]. Correlations between rock acceleration and PGA were established for each of the 68 top-layer grid cells using real-time seismic load data. Consequently, PGAs for the two earthquake scenarios were mapped onto a 2D satellite image (Figure 10). For the hypothetical Pohang earthquake scenario, approximately 80% of the target building’s spatial grid cells exhibited PGAs exceeding 0.11 g. In contrast, during the actual Pohang earthquake, most grid cells (except for the central zone) exhibited PGAs below 0.01 g [33].

**Figure 11:**
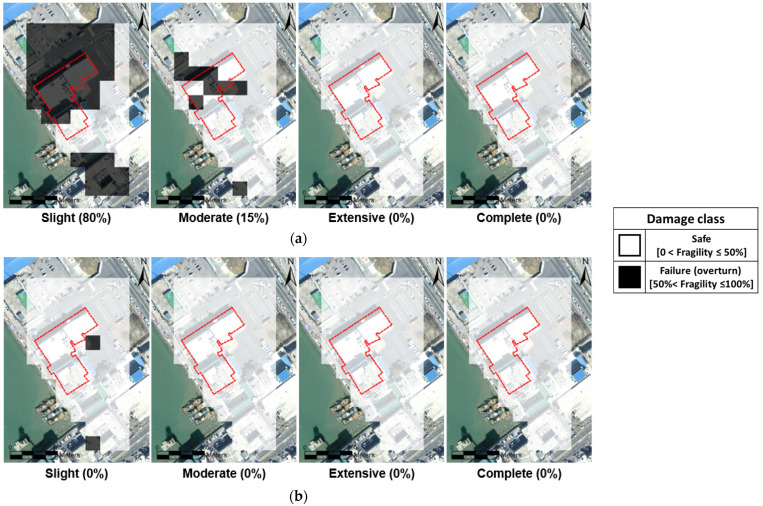
Structure fragility zonation maps of four damage levels for the target port for two earthquake scenarios. (**a**) Hypothetical Pohang earthquake scenario and (**b**) Pohang earthquake event.

Structural fragilities were automatically estimated using the real-time framework for seismic failure evaluation. Fragility curves (Figure 4) were linked to the PGAs derived from the geotechnical spatial grid. The failure probabilities for the four damage levels of unreinforced concrete structures were calculated based on the spatially distributed PGAs. Figure 11 illustrates the damage classification of the geotechnical spatial grid based on fragility curves.

For the hypothetical Pohang earthquake, 14 out of 17 spatial grid cells covering the passenger terminal’s ground surface were classified as having a ‘Slight’ failure state. The eastern part of the study area, covered by 18 grid cells, was deemed structurally safe due to the presence of non-amplifiable boulder stone or dredged silty clay. The overall passenger terminal was determined to have an 8% seismic failure probability, as 80% of its occupied grid cells were evaluated as having a ‘Slight’ damage state (Figure 11a).

In contrast, for the actual Pohang earthquake, all grid cells were evaluated as ‘Safe’, with fragility levels below 50%. As such, the passenger terminal was determined to be safe from seismic fragility (Figure 11b). A simple safety analysis confirmed that the structure would not experience significant failure under the Pohang earthquake scenario. Using the geotechnical spatial grid to account for site-specific seismic ground amplification, damage types such as differential settlement can be identified based on the zonal failure probabilities. This approach is potentially beneficial for immediate stabilization efforts, post-earthquake damage assessment, and structural safety evaluations.

## 4. Discussion

The purpose of this study was to develop and validate a real-time assessment framework for the seismic fragility of structures, applied to Incheon Port in South Korea. This framework integrates geotechnical spatial grid construction, real-time seismic load determination, and structure fragility evaluation using a GIS platform. The findings offer several key insights and implications for seismic hazard management and structural safety assessment.

### 4.1. Interpretation of Key Findings

The results demonstrate that the proposed framework effectively assessed the structural fragility of the passenger terminal under both real and hypothetical earthquake scenarios. For the hypothetical Pohang earthquake, the majority of the study area was classified as being in a “Slight” damage state, while the real Pohang earthquake scenario showed no significant structural damage. These findings suggest that the local site-specific conditions, including soil properties and seismic amplification effects, play a crucial role in determining the seismic vulnerability of the structure.

The application of kriging interpolation and geotechnical spatial grids provided a comprehensive understanding of the spatial variation in seismic hazards. By accounting for bedrock depth and soil conditions, the framework captured the amplification effects accurately, leading to more reliable fragility predictions. This highlights the importance of incorporating site-specific geotechnical data into seismic fragility assessments for coastal infrastructure.

### 4.2. Implications for Structural Safety and Seismic Risk Management

The framework’s ability to generate real-time fragility hazard maps has significant implications for disaster preparedness and risk management. These maps can be used to identify high-risk zones and prioritize structural retrofitting or stabilization efforts. Furthermore, real-time assessments can support rapid decision-making during seismic events, aiding in immediate evacuation planning and post-disaster recovery operations.

The classification of failure probabilities into different damage levels (e.g., “Slight”, “Moderate”, “Extensive”, and “Complete”) enables more precise structural safety evaluations. This approach can also help policymakers and engineers develop more targeted seismic mitigation strategies based on the specific vulnerabilities of each structure.

### 4.3. Strengths of the Proposed Framework

One of the primary strengths of this framework is its integration of geotechnical, seismic, and structural data in a GIS environment. This integration allows for real-time updates and visualization of seismic fragility across spatially distributed infrastructures. The use of kriging interpolation further enhances the accuracy of seismic hazard assessments by mitigating spatial uncertainties.

Another strength lies in the adaptability of the framework to various earthquake scenarios. By incorporating both real and hypothetical events, the framework can be calibrated for different seismic intensities and soil conditions, ensuring its robustness across diverse geotechnical environments.

### 4.4. Limitations and Future Directions

Despite its strengths, the study has several limitations that should be addressed in future research. First, the accuracy of the proposed framework heavily relies on the availability and quality of geotechnical and seismic data. Limited borehole data or low-resolution satellite imagery may reduce the reliability of fragility predictions. Expanding the dataset through more extensive ground surveys and integrating high-resolution spatial data—such as UAV- or LiDAR-based measurements—could significantly enhance the spatial resolution and precision of the framework, thereby improving the reliability and applicability of seismic risk assessments in regions lacking sufficient geotechnical information.

Second, the fragility functions utilized in this study were derived from general empirical models. While these models are widely employed and offer useful preliminary assessments, they may not fully capture the unique structural attributes of specific facilities, such as those at Incheon Port. To overcome this, future research should prioritize developing or calibrating site-specific fragility curves tailored to the detailed structural characteristics of individual buildings. This could be achieved through advanced numerical modeling or targeted experimental validation, ultimately enabling more accurate loss estimation and better-informed mitigation strategies.

### 4.5. Future Applications

The proposed framework has strong potential for extension to other critical infrastructure systems, including bridges, tunnels, and coastal industrial complexes. Notably, components of the modular GIS-based system have already been partially implemented in LNG terminals and KTX rail networks in Korea, demonstrating its adaptability to diverse operational contexts. Future integration with machine learning algorithms could further enhance the framework by enabling automated fragility predictions derived from both real-time event data and historical structural performance records.

In addition, linking this framework to national disaster response networks or early warning systems would substantially improve regional resilience. Such integration would facilitate proactive decision-making and optimized resource allocation before, during, and after seismic events.

It is also worth noting that the present study primarily focused on single seismic events, without explicitly addressing the effects of aftershocks or cumulative structural damage from multiple events. Incorporating multi-event sequences and applying time-dependent reliability analyses could provide a more realistic representation of cumulative damage processes and long-term resilience.

Finally, the robustness of the framework under scenarios involving sensor malfunctions or communication failures during extreme events remains to be examined. Future research should address these limitations by developing effective fail-safe protocols and redundancy strategies to reduce the risks associated with data acquisition disruptions. Such enhancements would improve the reliability, continuity, and operational readiness of structural monitoring systems in critical situations.

## 5. Conclusions

A systematic framework for the real-time assessment of structural seismic fragility was developed to account for local site response characteristics. The framework incorporates three interrelated assessment procedures in a real-time database: geotechnical spatial grid construction, real-time seismic load determination, and real-time structural fragility evaluation. First, a geotechnical spatial grid is constructed using 3D kriging interpolation of geotechnical data to represent the 3D seismic ground conditions, which are then correlated with the structure’s fragility function. Second, previously derived correlation equations between peak ground acceleration (PGA) and rock outcrop acceleration are integrated into the geotechnical spatial grid to reflect site-specific response characteristics. These correlated PGAs are linked to the fragility functions, allowing for the calculation of failure probabilities. The seismic damage grades of superstructures are then determined based on these failure probabilities. The proposed framework was applied specifically to Incheon Port in South Korea, using both actual Pohang earthquake data and a hypothetical earthquake scenario on a GIS platform. The simulation results were visualized in the form of a structural fragility hazard map, demonstrating the applicability and effectiveness of the computer-aided real-time assessment framework.

## Figures and Tables

**Table 1:** Earthquake event information [30].

Magnitude	5.4
Date and Time (KST)	15 November 2017, 14:29:31
Location (latitude, longitude)	36.11, 129.37
Depth (km)	7.0
Pohang earthquake event: recorded rock outcrop acceleration (g)	0.032 g
Hypothetical Pohang earthquake scenario: attenuated rock outcrop acceleration (g)	0.138 g

**Table 2:** Definition of damage levels of seismic fragility curves for the unreinforced concrete structure [4,29].

Damage Level	Slight	Moderate	Extensive	Complete
$\mathrm{Mean}\;\mathrm{value}\;(\bar{s}$)	0.2	0.4	0.8	1.6
Standard deviation (β)	0.64	0.67	0.66	0.66
Damage State	8%	40%	80%	100%

## Data Availability

The data supporting the findings of this perspective paper are available from the corresponding author upon reasonable request.
